# Dynamic Reorganization of the Cytoskeleton during Apoptosis: The Two Coffins Hypothesis

**DOI:** 10.3390/ijms18112393

**Published:** 2017-11-11

**Authors:** Suleva Povea-Cabello, Manuel Oropesa-Ávila, Patricia de la Cruz-Ojeda, Marina Villanueva-Paz, Mario de la Mata, Juan Miguel Suárez-Rivero, Mónica Álvarez-Córdoba, Irene Villalón-García, David Cotán, Patricia Ybot-González, José A. Sánchez-Alcázar

**Affiliations:** 1 Centro Andaluz de Biología del Desarrollo (CABD), and Centro de Investigación Biomédica en Red: Enfermedades Raras, Instituto de Salud Carlos III, Consejo Superior de Investigaciones Científicas, Universidad Pablo de, Carretera de Utrera Km 1, 41013 Sevilla, Spain; sulevapovea@gmail.com (S.P.-C.); manueloropesa@hotmail.com (M.O.-Á.); patricia_dlcruz_ojeda@hotmail.com (P.d.l.C.-O.); marvp75@gmail.com (M.V.-P.); mrdelamata@gmail.com (M.d.l.M.); juasuariv@gmail.com (J.M.S.-R.); monikalvarez11@hotmail.com (M.Á.-C.); villalon.irene@gmail.com (I.V.-G.); lobolivares@hotmail.com (D.C.); 2 Grupo de Neurodesarrollo, Unidad de Gestión de Pediatría, Instituto de Biomedicina de Sevilla (IBIS), Hospital Universitario Virgen del Rocío, 41013 Sevilla, Spain; pachybot@yahoo.co.uk

**Keywords:** apoptosis, apoptotic microtubule network, microtubules, actin filaments, genotoxic drugs

## Abstract

During apoptosis, cells undergo characteristic morphological changes in which the cytoskeleton plays an active role. The cytoskeleton rearrangements have been mainly attributed to actinomyosin ring contraction, while microtubule and intermediate filaments are depolymerized at early stages of apoptosis. However, recent results have shown that microtubules are reorganized during the execution phase of apoptosis forming an apoptotic microtubule network (AMN). Evidence suggests that AMN is required to maintain plasma membrane integrity and cell morphology during the execution phase of apoptosis. The new “two coffins” hypothesis proposes that both AMN and apoptotic cells can adopt two morphological patterns, round or irregular, which result from different cytoskeleton kinetic reorganization during the execution phase of apoptosis induced by genotoxic agents. In addition, round and irregular-shaped apoptosis showed different biological properties with respect to AMN maintenance, plasma membrane integrity and phagocyte responses. These findings suggest that knowing the type of apoptosis may be important to predict how fast apoptotic cells undergo secondary necrosis and the subsequent immune response. From a pathological point of view, round-shaped apoptosis can be seen as a physiological and controlled type of apoptosis, while irregular-shaped apoptosis can be considered as a pathological type of cell death closer to necrosis.

## 1. Introduction: An Overview of Apoptosis

The term “apoptosis” was coined by Kerr et al. in the early 1970s to describe the ultrastructural features of dying cells seen during development of hepatocytes [1]. This has given rise to the concept that these cells have an intrinsic suicide program that predetermines their fate. Nowadays, apoptosis is conceived as the major type of programmed cell death (PCD) in multicellular organisms, being distinct but connected to other types of PCD like autophagy or programmed necrosis [2]. It is characterized by several morphological and biochemical features which take place while membrane integrity is maintained [3]. The typical hallmarks of apoptosis include cell shrinkage, convolution of the nuclear and cellular outlines, cell membrane blebbing, formation of apoptotic bodies, chromatin condensation, caspase activation and DNA fragmentation [4]. 

Animal development and tissue homeostasis depend on the appropriate regulation of apoptosis. It is involved in sculpting and deleting structures, morphogenesis, regulation of cell number and elimination of aberrant cells [5]. Paradoxically, cell death turns out to be essential in the proliferative environment of development through the release of factors that influence cell division and survival of adjacent tissues. It has been shown to be necessary during different stages of embryonic growth, including the mammalian blastocyst stage, when both the inner cell mass and the trophectoderm undergo apoptosis. However, apoptosis levels must be strictly programmed in order to maintain embryonic homeostasis [6]. Apoptosis also contributes to the formation of vesicles and tubes (e.g., neural tube) when epithelial sheets invaginate and tissue inside has to be eliminated [7]. Neurons and oligodendrocytes which are overproduced during the development of the nervous system are also eliminated by apoptosis [8]. One example of the role of apoptosis can be seen during palate fusion. Here, unbalanced apoptosis has been shown to be a cause for cleft palate, one of the most common oral malformations [9]. 

However, most of the knowledge that we have today about the regulation of PCD comes from three model organisms: *Caenorhabditis elegans*, *Drosophila melanogaster* and the mouse. In *C. elegans*, 131 of the 1090 final somatic cells undergo programmed cell death during embryogenesis [10]. Programmed cell death is an intrinsic characteristic of somatic cells, strictly controlled by cell lineage. Ablation of cell death genes *ced-3* and *ced-4* prevents apoptosis in cells that normally die. Instead, these cells survive and differentiate. The concept of apoptosis extends in *Drosophila*, as it is controlled by both environmental and genetic factors. During the developmental stage of *Drosophila*, PCD is present from the embryo stage until oogenesis [11]. The regulation of apoptosis in vertebrates appears considerably more complex and vast numbers of cells undergo apoptosis throughout development and tissue homeostasis in adulthood [6]. In humans, perturbations of the signalling cascades regulating apoptosis can result in a wide variety of human diseases such as cancers, infectious diseases including AIDS (Acquired Immune Deficiency Syndrome), autoimmune diseases and neurodegenerative diseases [12]. 

Apoptotic cell death develops in three distinct phases: induction, execution and clearance of the dying cell. The fate of apoptotic cells in multicellular organisms is their immediate elimination by phagocytes. However, cells that perform apoptosis in in vitro cultures progress to secondary necrosis, a process which entails the loss of membrane integrity and the release of cellular content into the surrounding interstitial tissue [13]. In vivo, secondary necrosis is also likely to happen in case of extensive cell death or impaired phagocytosis and it has been hypothesized to participate in the genesis of many human diseases [14]. 

The phase of induction encompasses all the intrinsic or extrinsic environmental changes that lead to the activation of the apoptotic signalling. Following induction, the execution phase takes place thanks to the activation of a caspase-dependent proteolytic cascade [15]. Caspases are aspartic acid-specific proteases responsible for cellular component degradation. Some of them, like caspase-8 and -9, act as initiators of the apoptotic signalling pathway, while other caspases like caspases-3, -6 and -7, operate as executor caspases which actively participate in the degradation of cell substrates [16]. Caspase activation can be initiated by two main apoptotic pathways, the extrinsic or death receptor pathway and the intrinsic or mitochondrial pathway. However, there is evidence that the two pathways are interconnected and that molecules in one pathway can influence the other [17]. Eventually, the dying cell is engulfed by professional phagocytes or by neighbouring cells. Efficient apoptotic cell removal is driven by the interaction with phagocytes through the expression of “eat-me” signals and the release of “find-me” signals, which facilitate the engulfment of the dying cell and its eventual digestion in their phagolysosomes. This process of apoptotic cell clearance is essential for tissue turnover and homeostasis [18]. In fact, this interaction prevents undesired immune reactions by contributing to the development of an immunomodulatory environment [19]. 

## 2. Genotoxic Cell Response and Cytoskeleton 

In the context of human disease, cancer is one of the most outstanding pathologies, in which apoptosis plays a major role. Evading apoptosis has been shown to be a hallmark of cancer as tumour progression is linked, not only to cell proliferation, but also to death insensitivity [20]. Despite being one of its main causes, apoptosis has been traditionally used as a target for cancer treatment [21]. Typical therapies involve genotoxic drugs (chemotherapy or ionising radiation) with the aim of targeting cell proliferation [22]. Many of the cytotoxic agents commonly used to treat cancer patients such as alkylating agents, platinum drugs, antimetabolites, topoisomerase poisons and ionising radiation cause high levels of DNA damage [23]. However, to prevent the transmission of damaged DNA during cell division, cells activate the DNA damage response (DDR) which depends on DNA damage repair pathways as well as cell cycle checkpoint activation to arrest the cell cycle [24]. If DNA damage is irreparable cells may signal for senescence (growth arrest), apoptosis or other pathways leading to cell death [25]. The DDR enables cells to detect damage, recruit multi-protein complexes at these foci and activate downstream signalling [26]. Depending on the extent of DNA damage, the DDR distinguishes between repairable and non-repairable DNA damage, and controls different cellular responses such as transient cell cycle arrest and DNA repair, senescence or cell death [27].

Central components of the DDR machinery are the phosphoinositide 3-kinase related kinases ATM (Ataxia-telangiectasia-mutated) and ATR (ataxia telangiectasia and Rad3-related). ATM responds mainly to double-strand break (DSBs), whereas ATR is activated by single-strand break (ssDNA) and stalled replication forks [28]. When ATM and ATR are recruited to sites of damage, they target many substrates, including downstream kinases such as checkpoint kinases Chk2 and Chk1, regulatory proteins such as p53, and scaffolding proteins such as BRCA1 and BRCA2 (breast cancer 1 and 2). Once these proteins are activated, they regulate the function of downstream effector proteins such as p21, Cdc25A and cyclin-dependent kinases (CDKs). Phosphorylation of p53 at serine 46 serves as a pro-apoptotic mark that induces the transcription of apoptotic genes such as BAX (BCL2 Associated X), PUMA (p53 upregulated modulator of apoptosis), NOXA and p53AIP1 (p53-regulated apoptosis-inducing protein 1) that finally activate the cell death pathway via the mitochondrial, intrinsic pathway [29]. However, p53 can also act in a transcription-independent mode targeting mitochondria and inducing BAX activation and mitochondrial outer membrane permeabilization (MOMP) [30].

The DDR response also includes cytoskeleton reorganizations. Thus, following DNA damage RhoA (Ras homolog gene family, member A) specific guanine nucleotide exchange factor (GEFs) such as neuroepithelioma transforming gene 1 (Net1) get activated [31], leading to a Fen1 dependent activation of the RhoA/ROCK (Rho-associated protein kinase) axis [32], which controls the organization of the actin cytoskeleton [33]. Net1 is a RhoA specific GEF that is frequently overexpressed in human cancer [34]. It has been reported that DNA damage activates Net1 to control RhoA- and p38 MAPK-mediated cell survival pathways in response to DNA damage [35]. In adherent cells, the cellular response to DNA damage involves Net1 dephosphorylation and translocation from the nucleus to the cytosol where it activates RhoA GTPase [36]. In turn, Rho A activation controls actin filaments reorganization through the activation of ROCK and MLC (myosin light chain) phosphorylation and actinomyosin contractility [37]. Knock down of Net1 by RNAi prevents RhoA activation, inhibits the formation of stress fibres and enhances cell death [36]. This indicates that Net1 activation is required for RhoA mediated response to genotoxic stress and that cytoskeleton reorganization may play an important role in DDR. The Net1 and the RhoA dependent signals also converge in the activation of mitogen-activated protein kinase p38 (p38 MAPK) and its downstream target MAPK-activated protein kinase 2 (MK2) [36].

The importance of cytoskeleton reorganization during DDR and its role in genotoxic resistance or apoptosis induction is not completely understood and needs further research. 

## 3. Cytoskeleton Rearrangements during the Execution Phase of Apoptosis

The execution phase of apoptosis is denoted by cell contraction, plasma membrane blebbing, chromatin condensation and DNA fragmentation [38]. To achieve such dramatic morphological changes, apoptotic cells make profound cytoskeleton reorganizations, and caspase-mediated digestion of cytoskeleton proteins ensures the proper dismantlement of the dying cell during this process [39]. 

**Figure 1:**
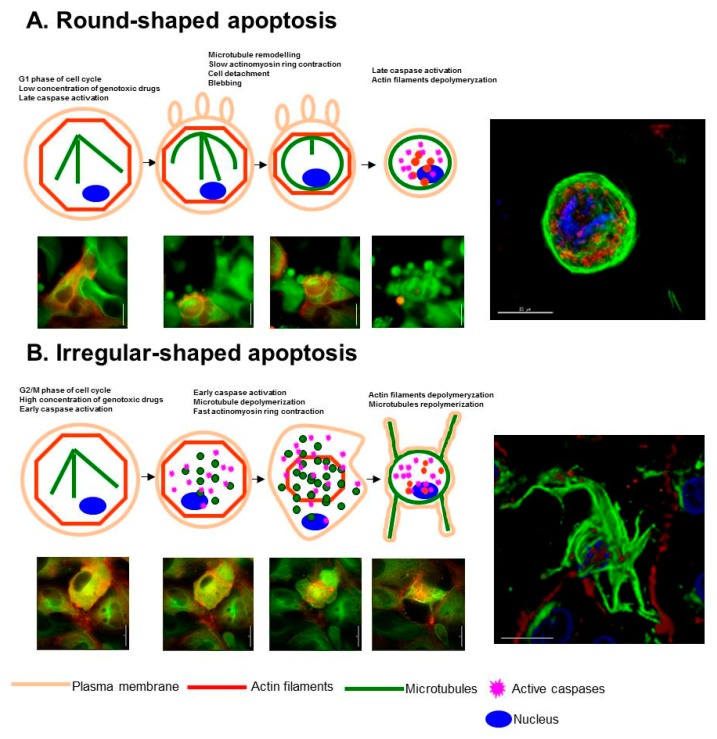
Schematic representation of the reorganization of actin filaments and microtubules during round (**A**) and irregular (**B**) -shaped apoptosis. Brown = plasma membrane; Blue = nucleus; Green = microtubules; Red = actin filaments; Pink = active caspases. Representative sequential images of round and irregular-shaped apoptosis in LLCPK-1α cells expressing GFP-αtubulin and pdsRed-monomer-actin are also included. Apoptosis was induced by camptothecin treatment. Right panels, immunofluorescence microscopy of round and irregular H460 apoptotic cells. Green = anti-α-tubulin: Red = anti-actin; Blue = Hoechst staining for nuclei. Scale bar= 15 µm.

The eukaryotic cytoskeleton is mainly composed of actin filaments, microtubules and intermediate filaments. These three constituents act coordinately to increase tensile strength, allow cell motility, maintain plasma integrity, participate in cell division, contribute to cell morphology and provide a network for cellular transport [40]. Classically, it has been accepted that microtubules and intermediate filaments are disorganized at the onset of the execution phase [38], while the actin cytoskeleton is responsible for cell remodelling during this phase [41]. At later stages, it has been observed that microtubules are reorganized [42,43], giving rise to the apoptotic microtubule network (AMN), a structure that sustains apoptotic cell morphology and maintains plasma membrane integrity [44,45] and participates in the dispersion of cellular and nuclear fragments [41,46]. However, this model has recently been expanded by new evidence that supports the hypothesis that genotoxic drugs induce two dose-dependent types of apoptosis characterized by different cytoskeleton rearrangement kinetics depending on caspase activation timing (Figure 1) [47]. Thus, “slow” or round-shaped apoptosis is characterized by late caspase activation, slow actinomyosin ring contraction, plasma membrane blebbing, cell detachment, microtubules remodelling, and formation of a round-shaped AMN and apoptotic cell morphology. In contrast, “fast” or irregular–shaped apoptosis is characterized by early caspase activation, initial microtubules depolymerisation, fast actinomyosin ring contraction without cell detachment, and formation of an irregular-shaped AMN and apoptotic cell morphology which frequently shows apoptotic membrane protrusions or microtubule spikes. Both round and irregular AMN have been observed during apoptosis induced by a variety of genotoxic agents (camptothecin, doxorubicin, teniposide and cisplatin) in several cell lines (H460, HeLa, MCF7 and LLCPK-1α) [47].

How do cells undergo round or irregular-shaped apoptosis after the exposition to apoptosis inducers such as chemotherapeutic compounds? In part, genotoxic agent concentration and cell cycle phase determine the cell response [47]. First, round and irregular AMNs are dependent on the concentration of the apoptotic stimulus. At low concentrations of genotoxic agent, cells undergo slow apoptosis and display a rounded AMN, whereas at higher concentrations, cells undergo fast apoptosis and show an irregular AMN. It is reasonable to infer that treatment with low doses of chemotherapeutic agents induces a slower cell death than that produced by high doses which can rapidly activate caspases by the intrinsic pathway. Second, apoptotic and AMN morphology are also dependent on the cell cycle phase. Thus, cells in G1 undergo round-shaped apoptosis while cells in G2/M undergo irregular-shaped apoptosis, irrespective of the concentration of the genotoxic agent [47]. Induction of tumour cell death by chemotherapeutic modalities often occurs in a cell cycle-dependent manner. Thus, it has been observed that several regulatory proteins involved in tumour chemosensitivity and apoptosis are expressed periodically during cell cycle progression [48,49,50]. Experimental studies have previously shown that apoptotic cell death can occur either fast (~min) or very slow (~h) [51]. Results from the Monte Carlo study also showed two types of apoptosis that can switch from slow (~h) to fast (~min), as the strength of an apoptotic stimulus increases [52]. Traditionally, slow apoptosis can be initially considered as a caspase-independent cell death in which caspases may be activated at late stages [53]. However, more research is needed to clarify the mechanisms behind the cell cycle dependency of cytoskeleton reorganization during apoptosis.

### 3.1. Reorganization of Microtubules during Apoptosis

Microtubules are polar protofilaments made up of α and β tubulin which are involved in supporting and maintaining the shape of cells, cell polarity and migration, intracellular transport of vesicles and organelles and segregation of chromosomes in mitosis [54]. 

The recent hypothesis of two types of apoptosis proposes that microtubules undergo two kinetically different processes (Figure 1) [47]. In round-shaped apoptosis, microtubules are not depolymerized at early stages of apoptosis. Instead, they are remodelled and acquire a concentric organization forced by the actinomyosin ring contraction. In round-shaped apoptosis, microtubule nucleation depends on the γ-tubulin ring complexes (γTuRC) which are not disorganized by caspases that are activated at later stages. In contrast, when caspases are activated at early stages during irregular-shaped apoptosis, γTuRC are degraded and interphase microtubules are soon disorganized [55,56,57]. This disassembly of microtubules is followed by a fast and full actinomyosin ring contraction. Next, actin filaments are depolymerised. In this situation, apoptotic cells are devoid of the main cytoskeletal elements. Soon after, microtubules are repolymerized adopting an irregular disposition beneath the plasma membrane [58]. 

**Figure 2:**
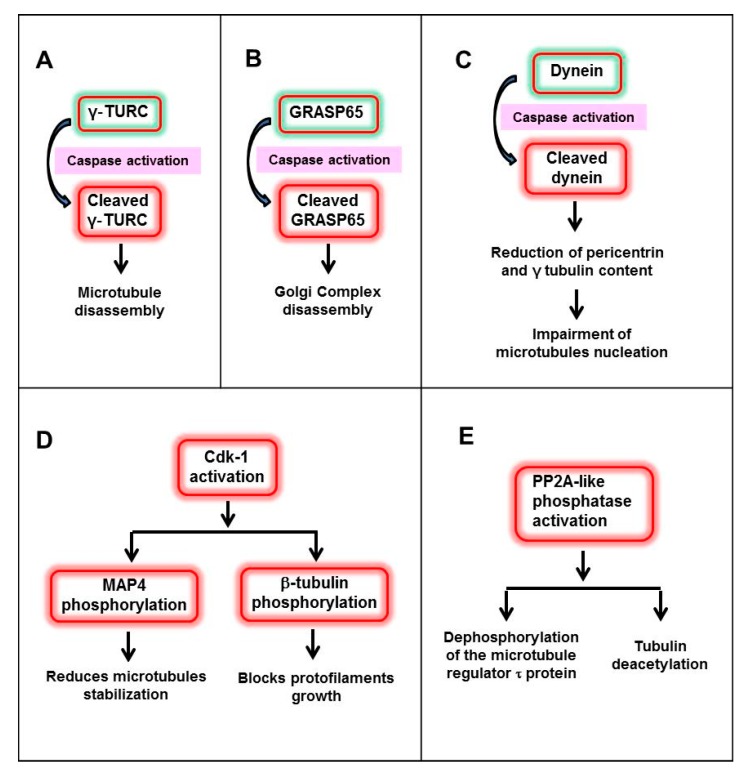
Molecular mechanisms involved in microtubules depolymerisation during the initial stages of irregular-shaped apoptosis: Cleavage of γ-TURC (**A**); cleavage of pericentriolar proteins such as GRASP65 (**B**); cleavage of dynein, a microtubule motor protein (**C**); activation of Cdk1, a kinase which regulates several microtubule effectors (**D**); and PP2A-like phosphatase activation which induces dephosphorylation of the microtubule regulator τ protein and tubulin deacetylation (**E**).

The molecular mechanisms involved in microtubule depolymerisation during the initial stages of irregular-shaped apoptosis are still unknown (Figure 2). One possibility is that early caspase activation can directly cleave γ-TURC provoking microtubule disassembly [47]. Alternatively, pericentriolar proteins could be targeted by caspases. Thus, GRASP65 (Golgi reassembly-stacking protein of 65 kDa), a pericentriolar protein related to the Golgi apparatus, has been described as a caspase target [59,60]. Another possible explanation relies on dynein, a microtubule motor protein, which is essential for the centrosomal localization of pericentrin and γ-tubulin in living cells [61]. Caspase cleavage of the dynein intermediate chain stops its motility and reduces the content of pericentrin and γ-tubulin at the centrosome, thereby impairing its capacity to nucleate microtubules [62]. Another hypothesis relies in the concept that microtubule dynamics are governed by several effectors such as motor proteins, gradients Ran-GTP, + ends proteins and microtubule-associated proteins (MAPs), which in turn are under the control of phosphatases and kinases [63,64,65]. One of the main kinases involved in phosphorylation is the cyclin-dependent kinase 1 (Cdk1), which associates with cyclin B as a key regulatory kinase that controls the entry into mitosis and regulates microtubule dynamics [66]. Cdk1 regulates some microtubule effectors by phosphorylation. For instance, MAP4 reduces its ability to stabilize microtubules after this modification [67]. In addition, Cdk1 is able to block protofilament growth during mitosis by phosphorylating β-tubulin [68]. Although Cdk1 activity is not apoptosis-specific, it has been observed during cell death. Therefore, it has been suggested that it may act as an essential regulator of microtubule reorganizations [57]. On the other hand, other authors have shown that microtubule depolymerisation at the onset of apoptosis is associated with activation of the PP2A-like phosphatase, dephosphorylation of the microtubule regulator Tau (τ) protein and tubulin deacetylation [69]. Both mechanisms can coexist in apoptosis as PP2A-mediated dephosphorylation of cdc25, a CdK1 regulator, precludes mitotic entry [70]. 

Once interphase microtubules are depolymerized and the actinomyosin ring contracts, apoptotic microtubules are reassembled during irregular-shaped apoptosis. The complete sequence of events that guide this repolymerisation are still undiscovered. However, it is known that irregular AMN is organized independently of γ-TURC, indicating that AMN formation is regulated by other mechanisms [47]. It has been hypothesized that active caspases may cleave the C-terminal regulatory region of tubulin during the execution phase, thereby increasing its ability to polymerize and thus facilitating the formation of apoptotic microtubules [60,71]. Another possible candidate for AMN nucleation could be core centrioles, which are not degraded during the execution phase. However, apoptotic microtubules are not displayed in the typical radial pattern of interphase microtubules, suggesting that centrioles are unlikely to guide the formation of AMN in irregular apoptosis.

Even though the mechanisms governing AMN arrangement in irregular apoptosis are unknown, this process seems to be tightly regulated. It has been proposed that the Ras-like small GTPase Ran could be responsible for this. Ran-GTP’s best known role focuses on regulating microtubule nucleation and dynamics during mitosis and meiosis [72]. Interestingly, Ran-GTP controls microtubule dynamics and motor activity [64]. It has been described that active Ran-GTP is necessary for apoptotic microtubule polymerization, and that its release into the apoptotic cytoplasm triggers microtubule nucleation [73]. Furthermore, RanGTP-activated spindle-assembly factor, TPX2 (targeting protein for Xklp2), escapes from the nucleus during the execution phase and associates with apoptotic microtubule and promotes their assembly [74]. Therefore, it has been hypothesized that apoptotic microtubule polymerization shares several mutual features with mitotic and meiotic spindle assembly, with a particular dependence on Ran-GTP and TPX2 [75].

After AMN reorganization, there is another level of regulation since apoptotic microtubules remain dynamic as it has been demonstrated by time-lapse imaging of the EB1 protein, a plus-end tracking protein [56,76]. 

### 3.2. Reorganization of Actin Cytoskeleton during Apoptosis

Unlike microtubules, actin cytoskeleton has been traditionally considered as a highly dynamic cytoskeletal element at the onset of apoptosis [58]. According to recent findings, actin cytoskeleton also suffers two kinetically different reorganizations during apoptosis (Figure 1). While round-shaped apoptosis depends on a slow contraction of the actinomyosin ring, irregular-shaped apoptosis is the result of a faster and full contraction. 

Slow or round-shaped apoptosis coincides with traditional cytoskeletal reorganizations described in apoptosis [39]. After apoptosis induction, adherent cells lose their focal adhesion sites and partially detach from their substrates. Then, actin filaments are reorganized beneath the plasma membrane into an actinomyosin cortical ring with contractile force (Figure 1). Characteristically, actinomyosin contraction produces plasma membrane blebbing which could travel away from the apoptotic cell and alert surrounding and immune cells before secondary necrotic membrane breakdown [77,78] .

**Figure 3:**
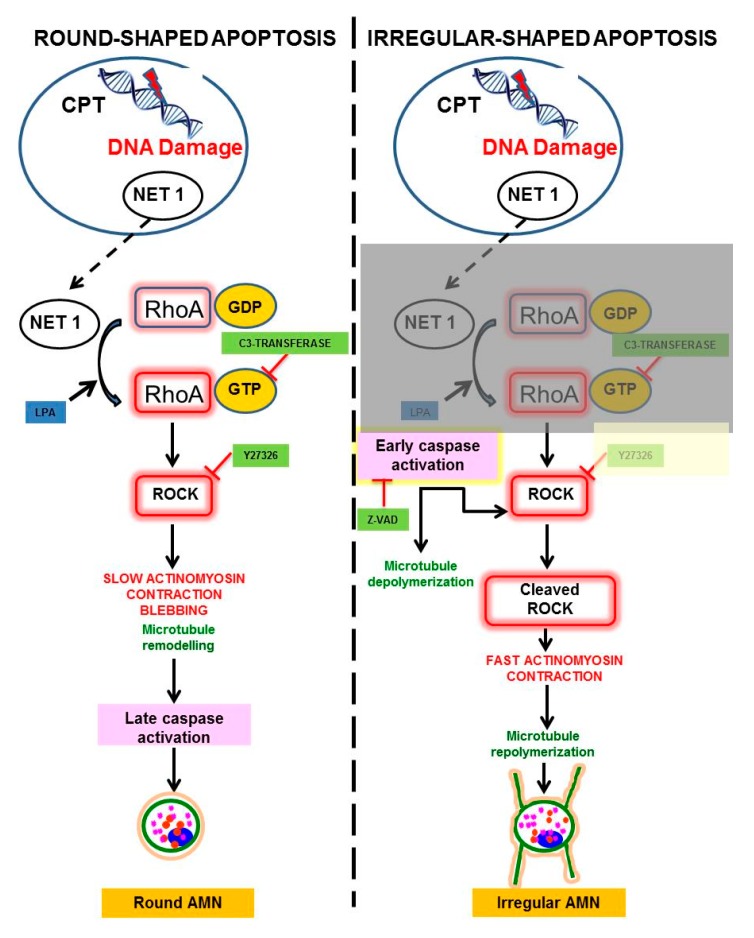
Schematic representation of signalling pathways involved in cytoskeleton reorganizations in round and irregular apoptosis. T arrow=inhibition; dashed arrow= translocation from nucleus to cytosol; solid arrow=activation; shaded area= disruption by caspases.

Actinomyosin contraction is activated via the RhoA/ROCK signalling pathway (Figure 3) [79]. RhoA GTPases are activated by interchanging GDP for GTP. This activation is controlled by Rho guanine-nucleotide-exchange factors (Rho GEFs), such as Net1, when cells are exposed to DNA damaging agents [31,75]. Net1 has been shown to localize preferentially within the nucleus at steady state [80]. Nuclear import of Net1 is mediated by two nuclear localization signals present in the N-terminus of the protein, and forced cytoplasmic localization of Net1 is sufficient to activate Rho. Net1 can move in and out of the nucleus, and the activation of RhoA by Net1 is controlled by changes in its subcellular localization. A logical prediction of this hypothesis is that in order for Net1 to be functionally active, it must be transported out of the nucleus into the cytosol, where it can activate RhoA. However, DNA damage signals such as ionizing radiation (IR), which has been previously shown to stimulate RhoA, specifically promoted the activation of the nuclear pool of RhoA in a Net1-dependent manner while the cytoplasmic activity was not affected [81].

Irrespective of the localization of RhoA activation by Net1, it activates its downstream effector, the Rho-associated coiled-coil protein kinase 1 (ROCK-1). Next, ROCK phosphorylates myosin light chain phosphatase (MLCP), which in turns increases the phosphorylation levels of myosin light chain II (MLC). As a consequence, contraction of the actinomyosin ring is induced [82]. This sequence of events can be considered as “slow” (hours) in comparison to irregular-shaped apoptosis (minutes). In summary, when caspases are not activated at early stages of apoptosis, actinomyosin ring contracts via the NET1/RhoA/ROCK/MLC phosphorylation pathway, causing a slow round-shaped apoptosis. As microtubules are not depolymerized, they are adjusted to the actinomyosin ring acquiring a circular organization. Later, when caspases are activated, the actinomyosin ring disappears and a round AMN remains in apoptotic cells. The reasons of why in this situation active caspases do not cleave γ-TURC and induce AMN depolymerisation are not known, although we can speculate that the concentric remodelling of microtubules during round-shaped apoptosis can make the access of caspases to γ-TURC difficult.

In contrast, early caspase activation in irregular apoptosis completely changes the kinetics of actin reorganization during apoptosis. First, as mentioned above, microtubules are depolymerized presumably by degradation of γ-TURC. Second, caspase degradation of NET 1 interferes with NET1/RhoA/ROCK/MLC phosphorylation/actinomyosin ring contraction pathway. Furthermore, caspase activation generates a constitutively active fragment of ROCK1 [83] that causes a rapid and full contraction of the actinomyosin ring and, as a result, the whole cell became a “big bleb” that remained attached to the substrate. Once actinomyosin contraction is finished, apoptotic cells are devoid of the main cytoskeletal elements and an irregular AMN is formed independently of γ-TURC. 

### 3.3. Reorganization of Intermediate Filaments during Apoptosis

Intermediate filaments that help maintain the integrity of tissues and cells are disrupted early at the onset of apoptosis by the action of caspases. The intermediate filament cleavage cause fragmentation and aggregation, and the breaking of the nuclear lamins facilitates nuclear disintegration [84,85,86].

The influence of intermediate filaments (IF) proteins on the cytoskeleton reorganization kinetics that lead to round of irregular-shaped apoptosis has not been investigated yet, so this review will only focus on the current knowledge of the role of microtubules and actin filaments during apoptosis. 

## 4. Modulation of Round and Irregular-Shaped Apoptosis

As irregular-shaped apoptosis is dependent on early caspase activation, inhibition of caspases by z-VAD (benzyloxycarbonyl-valine-alanine-aspartate-fluoromethylketone) prevents both microtubules depolymerisation and ROCK1 and NET1 cleavage, allowing a slow actinomyosin ring contraction and, consequently, apoptotic cells adopt a round-shaped morphology [47].

On the other hand, inhibition of actinomyosin ring contraction by C3-transferase, a RhoA inhibitor, or Y27632, a ROCK inhibitor, induce early caspase activation and apoptotic cells adopt an irregular morphology [47]. On the contrary, activation of actinomyosin ring contraction by lysophosphatidic acid (LPA), a RhoA activator, induces apoptotic cells with round-shaped AMN [47]. These findings indicate that round- and irregular-shaped apoptosis can be modulated by specific inhibitors or activators of the NET1-RhoA-ROCK-MLC pathway.

## 5. Biological Implications of the “Two Coffins” Model in Apoptosis

In a metaphorical sense, AMN can be considered as an intracellular “coffin” that protects the plasma membrane and confines the degradative processes of apoptotic cells. According to the proposed hypothesis, cells undergoing apoptosis can actually exhibit two types of “coffins” with kinetically different cytoskeleton reorganizations and distinctive properties with respect to resistance of apoptotic cells to undergo secondary necrosis and the ability of being phagocytosed.

The evaluation of the resistance of apoptotic cells to undergo secondary necrosis revealed that round-shaped apoptotic cells were more resistant to secondary necrosis than irregular apoptotic cells, consistent with a more homogeneous organization of apoptotic microtubules in apoptotic cells with round-shaped AMN [47]. This property of round-shaped apoptosis can be interesting for design therapies with low inflammatory responses. On the contrary, irregular-shaped apoptotic cells undergo secondary necrosis more easily, and consequently, they are more prone to induce inflammation.

The last stage of apoptosis comprises the elimination of apoptotic cells by macrophages or neighbouring cells. In the former case, clearance of apoptotic cells by phagocytes during efferocytosis can be divided into four distinct processes: recruitment of phagocytes near apoptotic cells; recognition of dying cells thanks to surface bridge molecules or receptors; engulfment of apoptotic cells; and degradation [87]. Efficient phagocytosis of apoptotic cells by macrophages depends on the presence of apoptotic microtubules [88]. Indeed, it has been shown that apoptotic cells with AMN show high expression of phosphatidylserine on the cell surface and increased phagocytosis rate. However, both processes were markedly reduced when AMN was depolymerized by colchicine treatment and cells undergo secondary necrosis. The ability of apoptotic cells to stimulate their phagocytosis by macrophages before cell lysis is crucial to prevent adverse effects, such as tissue damage and inflammation, associated with secondary necrosis [13]. As a consequence, phagocytosis reduces the probability of inflammation by ensuring that apoptotic cells are eliminated before the release of intracellular contents into the extracellular medium.

The intensity of externalization of phosphatidylserine is similar in both round- and irregular-shaped apoptosis [47]. Despite this, round apoptotic cells are more efficiently engulfed by professional phagocytes rather than irregular apoptotic cells [47]. These differences could be due to the different pattern of cytoskeleton organization and final apoptotic cell morphology. In addition, the process of blebbing, which takes place during round-shaped apoptosis, can be also important for efficient phagocytosis of apoptotic cells [78]. Accordingly, less blebbing capacity correlates with less engulfment efficiency. Hence, the inability of irregular-shaped apoptotic cells to undergo blebbing would explain why these cells show less phagocytosis potential. 

To evaluate whether rounded or irregular-shaped apoptosis became more pro-inflammatory, the levels of IL-1β in the medium from the phagocytosis assays were analysed [47]. IL-1β levels were significantly increased when macrophages were incubated with apoptotic cells with irregular morphology. However, IL-1β levels were not significantly increased in co-cultures with rounded apoptotic cells (which are more resistant to undergo secondary necrosis and more efficiently phagocytosed). These data suggest that irregular-shaped apoptosis may promote a higher production of pro-inflammatory cytokines by macrophages.

## 6. Conclusions and Future Perspectives

During the execution phase of apoptosis, the apoptotic microtubule network (AMN) adopts two different morphological patterns, round and irregular. Irrespective of different cytoskeletal rearrangements kinetics, AMN is required to maintain plasma membrane integrity and cell morphology during the execution phase of apoptosis. 

The NET1/RhoA/ROCK1/MLC phosphorylation/actinomyosin contraction signalling pathway operates when apoptosis is induced by low concentrations of genotoxic drugs, promoting round-shaped apoptosis. In contrast, early caspase activation in response to high concentrations of genotoxic drugs (that induces NET 1 and ROCK1 cleavage) disrupts this signalling pathway and promotes irregular-shaped apoptosis. 

Round- and irregular-shaped apoptosis are also dependent on cell cycle phase. Thus, cells in G1 undergo round-shaped apoptosis while cells in G2/M undergo irregular-shaped apoptosis, irrespective of the concentration of the genotoxic agent.

Round- and irregular-shaped apoptosis present different biological significance. Thus, round-shaped AMN makes apoptotic cells more resistant to secondary necrosis and less pro-inflammatory than irregular-shaped AMN. 

It would be interesting to explore whether these two types of apoptotic cells are present in different physiological or pathological situations, and whether they have distinct signalling roles or produce different types of signalling molecules. Furthermore, the knowledge and modulation of round and irregular apoptosis may be important for deciding better therapeutic options and predicting the subsequent immune response.
